# Suppression of NRF2 Activity by HIF-1α Promotes Fibrosis after Ischemic Acute Kidney Injury

**DOI:** 10.3390/antiox11091810

**Published:** 2022-09-14

**Authors:** Corry D. Bondi, Brittney M. Rush, Hannah L. Hartman, Jiaxuan Wang, Mohammad M. Al-Bataineh, Rebecca P. Hughey, Roderick J. Tan

**Affiliations:** Department of Medicine, University of Pittsburgh, Pittsburgh, PA 152671, USA

**Keywords:** acute kidney injury, HIF-1α, NRF2, ischemia, nutrient

## Abstract

Acute kidney injury (AKI) is a rapid decline in renal function and can occur after ischemia/reperfusion injury (IRI) to the tubular epithelia. The nuclear factor erythroid-2-related factor 2 (NRF2) pathway protects against AKI and AKI-to-chronic kidney disease (CKD) progression, but we previously demonstrated that severe IRI maladaptively reduced NRF2 activity in mice. To understand the mechanism of this response, we subjected C57BL/6J mice to unilateral kidney IRI with ischemia times that were titrated to induce mild to severe injury. Mild IRI increased NRF2 activity and was associated with renal recovery, whereas severe IRI decreased NRF2 activity and led to progressive CKD. Due to these effects of ischemia, we tested the hypothesis that hypoxia-inducible factor-1α (HIF-1α) mediates NRF2 activity. To mimic mild and severe ischemia, we activated HIF-1α in HK-2 cells in nutrient-replete or nutrient-deficient conditions. HIF-1α activation in nutrient-replete conditions enhanced NRF2 nuclear localization and activity. However, in nutrient-deficient conditions, HIF-1α activation suppressed NRF2 nuclear localization and activity. Nuclear localization was rescued with HIF-1α siRNA knockdown. Our results suggest that severe ischemic AKI leads to HIF-1α-mediated suppression of NRF2, leading to AKI-to-CKD progression.

## 1. Introduction

Acute kidney injury (AKI) is a rapid decline in renal function that is associated with high morbidity, mortality, and healthcare costs [1,2,3,4,5]. Survivors also have a higher risk of developing chronic kidney disease (CKD) and end-stage renal disease (ESRD) [6]. Treatments for AKI and AKI-to-CKD progression are urgently needed.

AKI can occur after ischemia/reperfusion injury (IRI), with tubular epithelial cells being particularly vulnerable [1,7]. The tubulointerstitial fibrosis that may occur following injury further exacerbates renal hypoxia [8]. To mitigate the effects of ischemic conditions, cells have evolved adaptive cytoprotective responses such as nuclear factor erythroid-2-related factor 2 (NRF2) and hypoxia inducible factor-1α (HIF-1α).

NRF2 is a transcription factor responsible for the upregulation of cytoprotective detoxifying and antioxidant genes [9]. It is regulated by a repressor, kelch-like ECH-associated protein 1 (KEAP1), which binds NRF2, and targets it for ubiquitin-mediated proteasomal degradation [10,11,12]. However, oxidative and electrophilic stress can inhibit KEAP1 and prevent NRF2 degradation. NRF2 accumulates and translocates to the nucleus where it binds to antioxidant response elements (AREs) to upregulate target genes, including NAD(P)H quinone dehydrogenase 1 (*NQO1*) [13,14].

Numerous studies demonstrate that NRF2 activation is protective in IRI-AKI. NRF2^−/−^ mice are markedly sensitized to IRI whereas pharmacologic activation of NRF2 protects against injury [15,16]. We and others show that mice with constitutive NRF2 activation are protected against AKI-to-CKD progression [17,18]. Interestingly, wild-type mice exposed to severe IRI exhibit a potentially maladaptive decrease in kidney NRF2 activity [17]. Similar reductions in NRF2 activity are seen in folic acid-induced nephropathy in mice and humans [19]. The mechanisms for reduced NRF2 activity are unknown.

HIF-1α is a transcription factor activated in tubular epithelial cells by hypoxia [20,21]. In normoxia, HIF-1α is hydroxylated by the prolyl hydroxylase domain-containing protein 2 (PHD2) and undergoes proteasomal degradation [22,23]. However, the activity of PHD2 is inhibited in hypoxic conditions, and HIF-1α accumulates and translocates into the nucleus where it forms a heterodimer with HIF-1β. The heterodimer then binds to hypoxia-response elements (HREs) to upregulate target genes including vascular endothelial growth factor (*VEGF*) [24,25]. Several studies show a protective role for HIF-1α in IRI-AKI. HIF-1α knockdown exacerbates ischemic injury in rodents and immortalized human proximal tubule epithelial cells (HK-2 cells) [26,27].

Because the activity of NRF2 and HIF-1α is simultaneously affected by IRI-AKI, we hypothesized that these pathways may crosstalk in the kidney. Studies show that HIF-1α reduces NRF2 activity or that NRF2 activates or inhibits HIF-1α [28,29,30,31,32,33,34,35], but these interactions have not been extensively studied in AKI. In this study, we demonstrate that the IRI severity determines whether NRF2 is activated or suppressed, and this effect is mediated by HIF-1α.

## 2. Materials and Methods

### 2.1. Animals

IACUC protocol approval was obtained at the University of Pittsburgh. Mice were afforded the ethical and scientific standards recommended by the Guide for the Care and Use of Laboratory Animals. The authors complied with the ARRIVE guidelines [36]. For the IRI study, 9- to 10-week-old (mean 24.1 g; range 21.7–27.3 g) male, wild-type C57BL/6J mice (#000664, Jackson Labs, Bar Harbor, ME, USA) were housed on a 12:12 h light–dark cycle with free access to water and Prolab^®^ IsoPro^®^ RMH 3000 5P75 diet. For the IOX2 study, 6- to 7-week-old (mean 23.8 g; range 21.9–25.7 g) male, wild-type C57BL/6J mice were provided the same conditions.

### 2.2. Antibodies

Primary antibodies utilized for immunoblot (IB), immunohistochemistry (IHC), and immunofluorescence (IF) were against ACTIN (IB: 1:10,000 #MAB1501, MilliporeSigma, Burlington, MA, USA), FIBRONECTIN (FN1) (IB: 1:1000, #F3648, MilliporeSigma), GAPDH HRP conjugated (GAPDH) (IB: 1:10,000, #HRP-60004, Proteintech, Rosemont, IL, USA), HIF-1α (IB: 1:1000, #14179S, Cell Signaling Technology, Danvers, MA, USA), NQO1 (IB: 1:1000, #ab2346, Abcam, Cambridge, UK), NQO1 (IB: 1:1000, IHC: 1:100, #11451-1-AP, Proteintech), NRF2 (IB: 1:1000, IF: 1:100, #16396-1-AP, Proteintech), TATA-binding protein (TBP) (IB: 1:1000, #44059S, Cell Signaling Technology), and VEGFA (VEGF) (IB: 1:1000, #26381-1-AP, Proteintech).

### 2.3. Ischemia/Reperfusion Injury

IRI was conducted according to modification of a published protocol [17]. Mice were randomly assigned to three groups of IRI severity: mild (15 min of ischemia), moderate (20 min of ischemia), and severe (30 min of ischemia). Briefly, mice were anesthetized with IP injection of ketamine (100 mg/kg) and xylazine (10 mg/kg), and surgeries were performed on a heating pad to maintain body temperature. After abdominal incision, the left renal pedicle was isolated, and an atraumatic surgical clamp placed (#RS-5459, Roboz, Gaithersburg, MD, USA). After confirming clamp placement, the kidney was observed for visible ischemia. The clamp remained for 15, 20, or 30 min before removal. Reperfusion was visually confirmed before closure. To assess renal function of the injured kidney, the contralateral kidney was removed 24 h prior to sacrifice or at day 10 for the day 28 endpoint. The mice were euthanized at 3 h or days 1, 3, 10, or 28 after clamp removal, at which time kidneys and sera were harvested. The total number of mice at time of sacrifice are as follows: Day 1, 15 min (3), 20 min (4), and 30 min (4); Day 3, 15 min (4), 20 min (4), and 30 min (4); Day 10, 15 min (3), 20 min (4), and 30 min (4); and Day 28, 15 min (2), 20 min (3) and 30 min (0). All three mice in the Day 28, 30 min group died after contralateral kidney removal on day 10.

### 2.4. In Vivo HIF-1α Induction

A 5 mg/mL N-((1,2-dihydro-4-hydroxy-2-oxo-1-phenylmethyl-3-quinolinyl)carbonyl)-glycine (IOX2;#11573, Cayman Chemical Company, Ann Arbor, MI, USA) stock was prepared with 100% DMSO. The stock was diluted with sterile Hank’s balanced salt solution (#14025-076, Gibco Laboratories) to 1 mg/mL. C57BL/6J mice were randomly assigned to either vehicle or IOX2 group. Mice were given two IP injections (37.7 mg/kg) 24 h apart [37]. Mice were euthanized 6 h after the last injection, and kidneys were harvested.

### 2.5. Serum Creatinine and Blood Urea Nitrogen

Serum creatinine level was assayed with the Creatinine (Enzymatic) Reagent Set (#C7548-120, Pointe Scientific, Canton, MI, USA). Blood urea nitrogen (BUN) level was assayed with the colorimetric QuantiChrom™ Urea Assay Kit (#DIUR-100, BioAssay Systems, Hayward, CA, USA).

### 2.6. Histology

Kidney tissue was fixed in 10% buffered formalin, paraffin embedded, and sectioned at 3 µm and stained with Periodic Acid Schiff (PAS) (MilliporeSigma) or Masson’s Trichrome Stain (MTS) (ThermoFisher Scientific, Waltham, WA, USA). The PAS images were subjected to pathologic scoring by an investigator (RJT) masked to group assignment according to the following scale: 0, no injury; 1, 1 to 25% of parenchyma affected by injury; 2, 26 to 50% involvement; 3, 51 to 75% involvement; and 4, 76 to 100% involvement. Injury was defined as tubular dilation, presence of casts, cell sloughing, or loss of brush borders.

### 2.7. Immunohistochemistry

Paraffin-embedded sections were hydrated through a series of xylene and graded alcohols. To the deparaffinized tissues, citrate-based antigen unmasking (Vector Laboratories, Burlingame, CA, USA) was performed followed by blocking for 1 h in 10% normal donkey serum before overnight incubation in primary antibody at 4 °C. Tissue was incubated in biotinylated secondary antibody (Jackson ImmunoResearch Laboratories, West Grove, PA, USA) for 2 h followed by a 1 h incubation in ABC reagent and developed with AEC reagent (both from Vector Laboratories). Slides were developed in the same batch and for the same amount of time.

### 2.8. Cell Culture

Immortalized human kidney proximal tubule epithelial cells (HK-2) (American Type Culture Collection, Manassas, VA, USA) were maintained in complete media ((Dulbecco’s modified Eagle medium/F12 (Gibco Laboratories, Gaithersburg, MD, USA) supplemented with 10% fetal bovine serum (Gibco Laboratories), and 100 U/mL penicillin and 100 µg/mL streptomycin (Gibco Laboratories) in a humidified atmosphere with 5% CO_2_ at 37 °C. Experiments were performed at 80% to 90% confluence.

HIF-1α activators, cobalt chloride (CoCl_2_) (Acros Organics, Morris, IL, USA) and IOX2 (Cayman Chemical Company), were used at concentrations indicated in the results [37,38,39,40]. HK-2 cells were exposed for 16 h to either nutrient-replete (complete media) or nutrient-deficient (Hanks’s balanced salt solution) conditions in the presence or absence of CoCl_2_ or IOX2 before lysing [26,41,42].

For HIF-1α knockdown experiments, cells were transfected with Lipofectamine^®^ RNAiMAX Reagent (#13778-075, Invitrogen, Carlsbad, CA, USA) and HIF-1α siRNA (#sc-35561, Santa Cruz Biotechnology, Dallas, TX, USA) or control siRNA-A (#sc-37007, Santa Cruz Biotechnology) in Opti-MEM^®^ medium (Gibco Laboratories) at a final siRNA concentration of 30 pM per well. The siRNA-lipid complex was added directly to the cell suspension. The cells were plated, and the media changed after 6 h. Twenty-four hours later, the cells were exposed to 200 µM CoCl_2_ in nutrient deficient conditions for 16 h before lysis.

### 2.9. Immunofluorescence

Cells were fixed in 2% paraformaldehyde for 30 min before quenching with 0.2 M NH_4_Cl for 10 min and permeabilization with 10% BSA in 0.1% Triton X-100 for 10 min. In a humidified chamber, cells were incubated overnight in primary antibody at 4 °C. Cells were incubated in fluorescent-labeled secondary antibody for 1 h at 4 °C. Imaging was performed by using a Leica TCS SP5 STED CW confocal microscope (Leica Microsystems Inc., Buffalo Grove, IL, USA).

### 2.10. Immunoblot

Tissue homogenates were prepared by douncing in pre-chilled radioimmunoprecipitation buffer (RIPA) supplemented with 1× Halt™ Protease & Phosphatase Single-Use Inhibitor Cocktail (ThermoScientific, Rockford, IL, USA) and centrifuged at 16,000× *g* for 15 min at 4 °C to recover the supernatant.

Whole-cell lysates were prepared by washing twice with ice-cold Dulbecco’s phosphate buffered saline (DPBS) containing CaCl_2_ and MgCl_2_ (Gibco Laboratories) before addition of pre-chilled RIPA buffer.

Subcellular fractionation was conducted by modification of a published protocol [43]. HK-2 cells were gently scraped in Buffer 1 (50 mM β-glycerophosphate, pH 7.3; 1.5 mM EGTA; 1 mM EDTA; 1 mM DTT; 1× Halt™ Inhibitor Cocktail) and centrifuged at 12,000× *g* for 5 min at 4 °C. Buffer 2 (20 mM HEPES, pH 7.5; 40 mM β-glycerophosphate, pH 7.3; 10 mM EGTA; 1 mM DTT; 1% Nonidet P40; 1× Halt™ Inhibitor Cocktail) was added to the pellet to lyse and centrifuged at 12,000× *g* for 5 min at 4 °C. The supernatant (cytosolic fraction) was saved. To this pellet (nuclear fraction), Buffer 3 (420 mM NaCl; 50 mM β-glycerophosphate, pH 7.3; 1.5 mM MgCl_2_; 0.2 mM EDTA; 1 mM DTT; 25% glycerol) was added and vortexed.

Protein concentrations were determined with the Pierce™ BCA Protein Assay Kit (ThermoScientific, Rockford, IL, USA). Samples were boiled in Laemmli sample buffer for 10 min. Equivalent protein concentrations were subjected to SDS-PAGE prior to transfer to PVDF membrane. The membrane was blocked in 5% nonfat milk and incubated overnight in primary antibody at 4 °C. The membrane was incubated in horseradish peroxidase-conjugated secondary antibody for 1 h before detection with Pierce™ SuperSignal^®^ WestPico Chemiluminescent Substrate (ThermoScientific).

### 2.11. Co-Immunoprecipitation

The cell extracts were centrifuged at 8000× *g* for 10 min at 4 °C. The supernatant was precleared by incubation for 20 min with 40 µL of 50% slurry of Protein G-conjugated to Sepharose (Invitrogen, Carlsbad, CA, USA) on ice before centrifugation at 15,300× *g* at 4 °C. The supernatant was incubated overnight in primary antibody (HIF-1α) at 4 °C. Protein G-conjugated to Sepharose (30 µL 50% slurry) was added with end-over-end mixing for 2 h at 4 °C. The immunoprecipitate on beads was washed once with 1 mL ice cold HEPES-buffered saline (150 mM NaCl; 10 mM HEPES, pH 7.4). Protein was eluted with 30 µL NuPAGE LDS 2x sample buffer (Invitrogen) including 10% reducing agent for 5 min at 90 °C. Cell extracts (1.5%) or immunoprecipitants were immunoblotted and developed with Bio-Rad Clarity ECL (Bio-Rad Laboratories, Hercules, CA, USA) and a ChemiDoc Imaging System (Bio-Rad Laboratories).

### 2.12. Quantitative, Real-Time Reverse Transcriptase Polymerase Chain Reaction

Total RNA was extracted with TRIzol^®^ Reagent (Ambion^®^, Carlsbad, CA, USA), and cDNA was generated with the RevertAid Reverse Transcriptase Kit (ThermoFisher Scientific, Pittsburgh, PA, USA). Reaction was performed by using iTAQ™ Universal SYBR^®^ Green Supermix (Bio-Rad Laboratories) and the CFX Connect™ Real-Time System (Bio-Rad Laboratories). Each reaction contained one of the following primer pairs: *β-ACTIN* NM_001101.5 (Human) (Forward: 5′-AGGCATCCTCACCCTGAAGTA-3′; Reverse: 5′-CACACGCAGCTCATTGTAGA-3′), *β-Actin* NM_007393.5 (Mouse) (Forward: 5′- CAGCTGAGAGGGAAATCGTG-3′; Reverse: 5′-CGTTGCCAATAGTGATGACC-3′), *Cat* NM_009804.2 (Mouse) (Forward: 5′-CAATGTCACTCAGGTGCG-3′; Reverse: 5′-CAGGGTGGACGTCAGTGAAA-3′), *Glut1* NM_011400 (Mouse) (Forward: 5′-CAGTTCGGCTATAACACTGGTG-3′; Reverse: 5′-GCCCCCGACAGAGAAGATG-3′) [44], *Gstm1* NM_001374678.1 (Mouse) (Forward: 5′-ATACTGGGATACTGGAACGTCC-3′; Reverse: 5′-AGTCAGGGTTGTAACAGAGCAT-3′), *Gstp1* NM_013541.1 (Mouse) (Forward: 5′-GGGAGCTGCCCATACAGAC-3′; Reverse: 5′-ATGCCACCATACACCATTGTC-3′), *HIF-1α* NM_001530.4 (Human) (Forward: 5′-GTTTACTAAAGGACAAGTCACC-3′; Reverse: 5′-TTCTGTTTGTTGAAGGGAG-3′) [26], *Kim1* NM_134248.2 (Mouse) (Forward: 5′- GGAATCCCATCCCATACTCCT-3′; Reverse: 5′-AAGTATGTACCTGGTGATAGCCAC-3′), *Ngal* NM_008491.1 (Mouse) (Forward: 5′-CCATCTATGAGCTACAAGAGAACAAT-3′; Reverse: 5′-TCTGATCCAGTAGCGACAGC-3′), *NQO1* NM_000903.3 (Human) (Forward: 5′-TGGTTTGGAGTCCCTGCCAT-3′; Reverse: 5′-CACTGCCTTCTTACTCCGGAAGG-3′), *Nqo1* NM_008706.5 (Mouse) (Forward: 5′-AGCCAATCAGCGTTCGGTAT-3′; Reverse: 5′- GCCTCCTTCATGGCGTAGTT-3′), *Pdk1* NM_172665.5 (Mouse) (Forward: 5′-GGACTTCGGGTCAGTGAATGC-3′; Reverse: 5′-TCCTGAGAAGATTGTCGGGGA-3′) [44], *Pgk1* NM_008828.3 (Mouse) (5′-GGAAGCGGGTCGTGATGA-3′; Reverse: 5′-GCCTTGATCCTTTGGTTGTTTG-5′) [45], *VEGF* NM_001025366.3 (Human) (Forward: 5′-CTACCTCCACCATGCCAAGT-3′; Reverse: 5′-GCAGTAGCTGCGCTGATAGA-3′), *Vegf* NM_001025257.3 (Mouse) (Forward: 5′-CCACGTCAGAGAGCAACATCA-3′; Reverse: 5′-TCATTCTCTCTATGTGCTGGCTTT-3′) [46]. Results were normalized to *β-Actin* and gene expression was determined with the 2^−ΔΔCt^ method [47]. Melt curve was performed to ensure specificity for a single product.

### 2.13. Statistical Analysis

A Shapiro–Wilk’s W normality test was performed. For two group comparisons, an unpaired, two-tailed Student’s *t*-test was performed. For comparisons between three or more groups, a one-way ANOVA was performed, followed by a Tukey’s multiple comparisons test. To assess between-group differences, a two-way ANOVA was performed followed by a Tukey’s or Dunnett’s multiple comparisons test. Results are reported as mean ± S.E.M. GraphPad Prism 7.03 software (GraphPad Software Inc., La Jolla, CA, USA) was used. The threshold for significance was *p* < 0.05.

## 3. Results

### 3.1. Severe Ischemia Promotes AKI-to-CKD Progression

**Figure 1:**
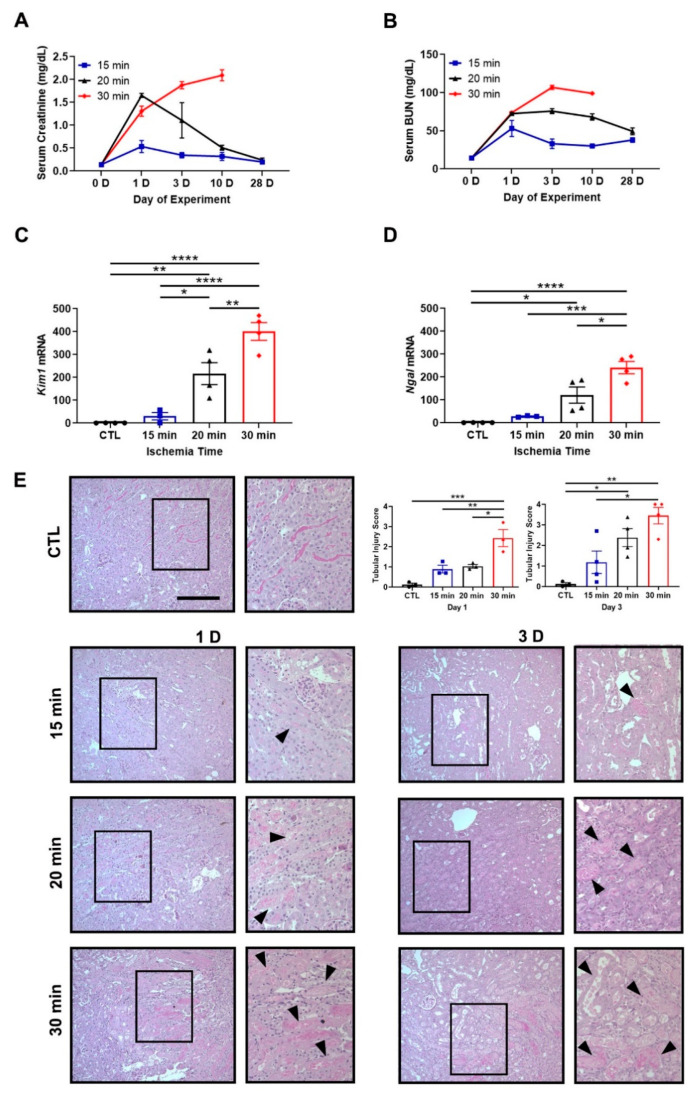
Ischemia time determines the magnitude of kidney injury after unilateral IRI. (**A**,**B**) Serum creatinine and blood urea nitrogen (BUN) levels at baseline and days 1, 3, 10, and 28. Thirty-minute ischemia leads to mice death by day 28. *n* = 2–4 mice/group. (**C**,**D**) *Kim1* and *Ngal* mRNA expression at day 10. *Kim1* and *Ngal* increase in the 20- and 30-min groups compared to control. The contralateral kidney from the 15-min group is used as the control. Bars are mean ± S.E.M. One-way ANOVA. * *p* < 0.05, ** *p* < 0.01, *** *p* < 0.001, and **** *p* < 0.0001. (**E**) Representative PAS-stained images are shown. Staining reveals increasing amounts of tubular damage with longer ischemia times (arrowheads). Contralateral kidney from day 1 is used as the control. Bar = 100 μm. Enlargement of the boxed area is shown to the right of each image. Included are tubular injury score at days 1 and 3. Bars are mean ± S.E.M. One-way ANOVA. * *p* < 0.05, ** *p* < 0.01, and *** *p* < 0.001.

C57BL/6J mice were subjected to unilateral IRI to evaluate AKI-to-CKD progression [17,48]. Clamp times of 15, 20, or 30 min were selected to induce mild, moderate, or severe IRI, respectively. Mild and moderate IRI led to a transient increase in serum creatinine and blood urea nitrogen (BUN) peaking at day 1. Severe IRI led to an inexorable rise in serum creatinine and BUN as well as death after contralateral kidney removal on day 10 (Figure 1A,B). At day 10, the mRNA expression of tubular injury markers, kidney injury molecule 1 (*Kim1*) and neutrophil gelatinase-associated lipocalin (*Ngal*), also increased with longer ischemia times (Figure 1C,D). Histology revealed a graded increase in tubular injury with longer ischemia times and was characterized by loss of brush borders, flattening of the epithelia, and cell death (Figure 1E).

**Figure 2:**
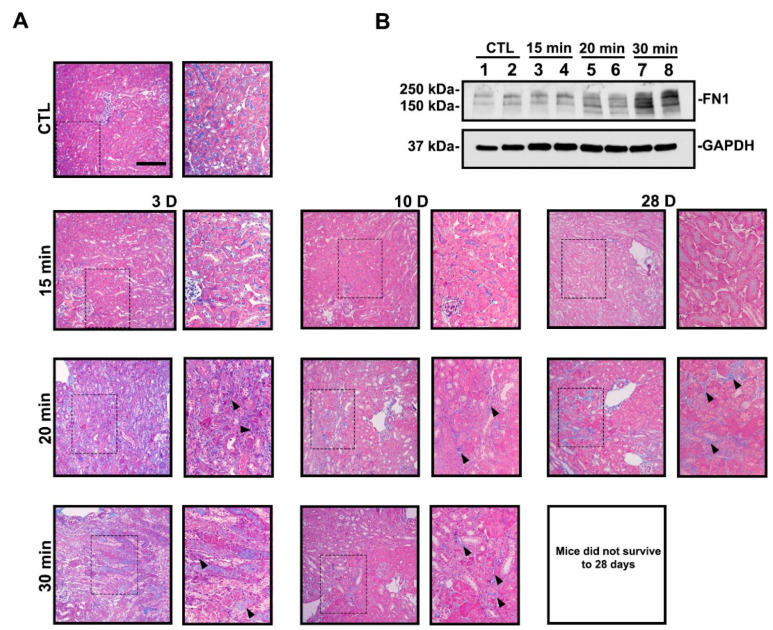
Longer ischemia times promote renal fibrosis. (**A**) Representative MTS images are shown. Thirty-minute ischemia leads to animal death by day 28. Kidney sections from the 30-min group display extensive collagen deposition (blue staining, arrowheads). Contralateral kidney from day 3 is used as the control. Bar = 100 μm. Enlargement of the boxed area is shown to the right of each image. (**B**) Immunoblot of FN1 protein expression at day 10. FN1 expression increased with longer ischemia times.

To assess AKI-to-CKD progression, we examined the development of renal fibrosis. Mice exposed to mild IRI did not exhibit fibrotic lesions on Masson’s trichrome stain, while longer ischemia times led to fibrosis (Figure 2A). As noted above, none of the mice exposed to severe IRI survived to day 28 after contralateral kidney removal on day 10. In agreement with our histology results, fibronectin (FN1) protein expression was increased with longer ischemia times on day 10 (Figure 2B, Supplemental Figure S1). Overall, mild IRI led to reversible AKI with minimal to no chronic disease, while moderate and severe IRI led to tubulointerstitial fibrosis, indicative of AKI-to-CKD progression.

### 3.2. IRI Severity Determines NRF2 and HIF-1α Activity

**Figure 3:**
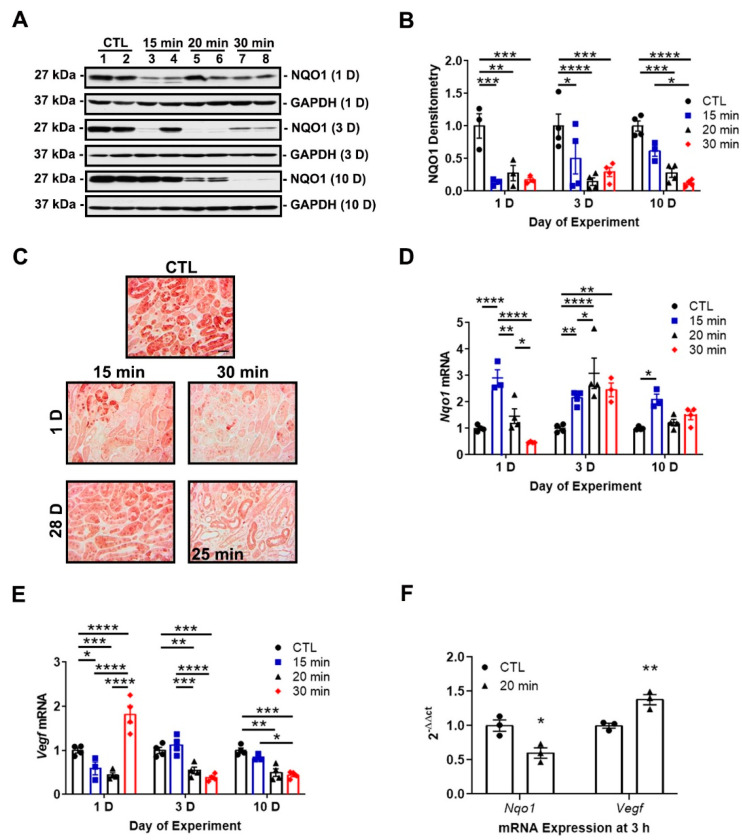
IRI severity determines NRF2 and HIF-1α activity. (**A**) Representative immunoblots of NQO1 protein expression at days 1, 3, and 10. NQO1 expression decreases at day 1 in all groups but recovers by day 10 in the 15-min group. Notably, NQO1 expression declines by day 10 in the 30-min group. (**B**) Densitometry of NQO1 normalized to GAPDH. (**C**) Representative IHC images of NQO1 protein expression at days 1 and 28. A surrogate 25-min group is substituted for the 30-min group in which there was no survival at day 28. Renal tubular NQO1 expression decreases at day 1 in both groups but recovers by day 28 in the 15-min group. Bar = 50 μm. (**D**,**E**) *Nqo1* and *Vegf* mRNA expression at days 1, 3, and 10. Sustained increases in *Nqo1* are induced by 15 min ischemia at all time points, while longer ischemia times lead to delayed and transient increases peaking at day 3. *Vegf* mRNA expression decreases in the 15- and 20-min groups but increases in the 30-min group at day 1. The 20- and 30-min groups show a sustained reduction in *Vegf* at days 3 and 10 compared to the control and 15-min groups. The contralateral kidney from the 15-min group is used as the control. (**F**) *Nqo1* and *Vegf* mRNA expression at 3 h after reperfusion. *Nqo1* decreases while *Vegf* increases in the 20-min group. Bars are mean ± S.E.M. Two-way ANOVA or unpaired Student’s *t*-test, as appropriate. * *p* < 0.05, ** *p* < 0.01, *** *p* < 0.001, and **** *p* < 0.0001.

Our previous study revealed a maladaptive decrease in NRF2 activity in wild-type mice during AKI, which we measured as a decrease in NQO1 protein expression [17]. We evaluated *Nqo1* expression over time in response to differing IRI severities. Supporting our prior results, immunoblot and immunohistochemistry analyses revealed decreased NQO1 protein expression at day 1 for all ischemia times. Although NQO1 expression recovered by day 10 in mild IRI, it declined in severe IRI (Figure 3A,C).

To determine the relationship of NRF2 and HIF-1α in progressive kidney injury, we examined mRNA expression of the NRF2 and HIF-1α targets genes of *Nqo1* and *Vegf*, respectively, over time in our mild, moderate, and severe IRI groups. HIF-1α and NRF2 activity are inversely correlated during AKI. Mild IRI led to an early (day 1) and sustained increase of *Nqo1* expression over the 10-day time course. Conversely, longer ischemia times led to delayed and transient increases in expression (Figure 3D). *Vegf* expression decreased in mild and moderate IRI at day 1 while *Nqo1* expression increased. However, severe IRI increased *Vegf* expression at day 1 while *Nqo1* expression decreased. At later time points, longer ischemia times led to decreased *Vegf* expression while *Nqo1* expression increased (Figure 3E). At an earlier time point, 3-h post-reperfusion, moderate IRI decreased *Nqo1* expression and *Vegf* expression increased (Figure 3F). Collectively, mild IRI led to rapid and sustained NRF2 activity and eventual restoration of NQO1 protein expression. However, in severe IRI, NRF2 activity is suppressed and associated with loss of NQO1 protein expression. HIF-1α activity could be responsible for the suppression of NRF2 activity.

### 3.3. NRF2 Activity Is Responsive to HIF-1α Activation and Nutrient Availability

**Figure 4:**
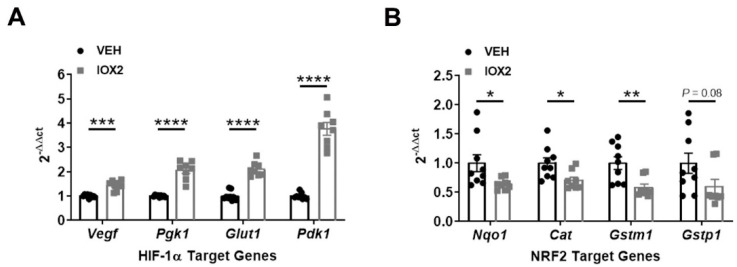
IOX2-mediated HIF-1α activation decreases NRF2 target genes. (**A**) HIF-1α target genes, *Vegf*, *Pgk1*, *Glut1*, and *Pdk1*, increase in response to IOX2 (37.7 mg/kg). (**B**) NRF2 target genes, *Nqo1*, *Cat*, *Gstm1*, and *Gstp1*, decrease in response to IOX2. Bars are mean ± S.E.M. Unpaired Student’s *t*-test. * *p* < 0.05, ** *p* < 0.01, *** *p* < 0.001, and **** *p* < 0.0001.

To examine the relationship between NRF2 and HIF-1α, we induced HIF-1α activation in mice with IOX2, a potent inhibitor of PHD2 [37], and assessed mRNA expression of HIF-1α and NRF2 target genes. IOX2-mediated HIF-1α activation increased mRNA expression of HIF-1α target genes while simultaneously decreasing expression of NRF2 target genes (Figure 4A,B). These results are consistent with HIF-1α mediating NRF2 activity.

**Figure 5:**
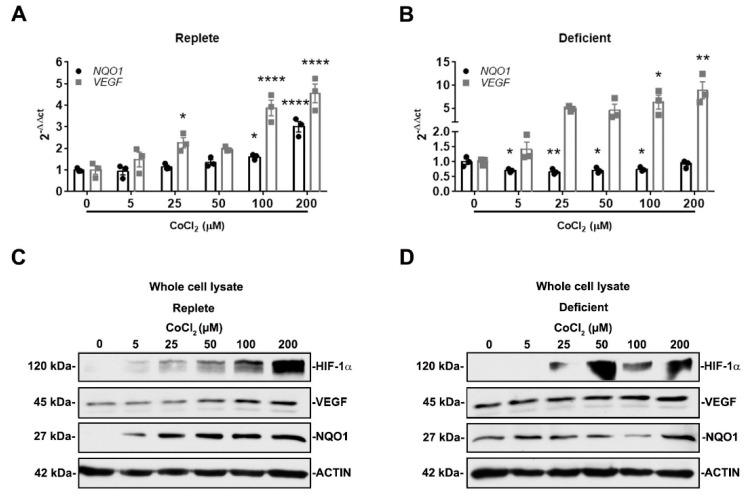
NRF2 activity is responsive to HIF-1α activation and nutrient availability. (**A**,**B**) *NQO1* and *VEGF* mRNA expression were examined after 16 h exposure to increasing concentrations of CoCl_2_ in either nutrient-replete or nutrient-deficient conditions. In nutrient-replete conditions, CoCl_2_ dose-dependently increases both *NQO1* and *VEGF* expression. In nutrient-deficient conditions, *VEGF* expression increases while *NQO1* expression decreases. (**A**,**B**) *n* = 3. Bars are mean ± S.E.M. One-way ANOVA. * *p* < 0.05, ** *p* < 0.01, and **** *p* < 0.0001 compared to condition without CoCl_2_. (**C**,**D**) Representative immunoblots of whole-cell lysates of HIF-1α, VEGF, and NQO1 protein expression after 16 h exposure to increasing concentrations of CoCl_2_ in either nutrient-replete or nutrient-deficient conditions. In nutrient-replete conditions, CoCl_2_ dose-dependently increases VEGF and NQO1 expression. However, in nutrient-deficient conditions, CoCl_2_ dose-dependently increases VEGF expression but not NQO1 expression.

To more fully understand how NRF2 and HIF-1α activity is affected by IRI severity in our model, we utilized an in vitro system that could mimic mild and severe IRI. Because ischemia impairs nutrient and oxygen delivery, we exposed HK-2 cells to either nutrient replete (complete media) or nutrient deficient (Hank’s balanced salt solution) conditions [26,41,42] in the presence or absence of HIF-1α activators, CoCl_2_ and IOX2 [37,38,39,40]. We postulated that HIF-1α activation in nutrient-deficient conditions would simulate a more severe injury compared to nutrient-replete conditions. In nutrient-replete conditions, CoCl_2_ dose-dependently increased both *VEGF* and *NQO1* mRNA and protein expression (Figure 5A,C). Interestingly, in nutrient deficient conditions, CoCl_2_ dose-dependently increased *VEGF* mRNA and protein expression but suppressed *NQO1* mRNA and protein expression (Figure 5B,D). These results are consistent with NRF2 activity being increased in mild IRI and decreased in severe IRI. This appears to be contingent on HIF-1α activation and nutrient availability.

**Figure 6:**
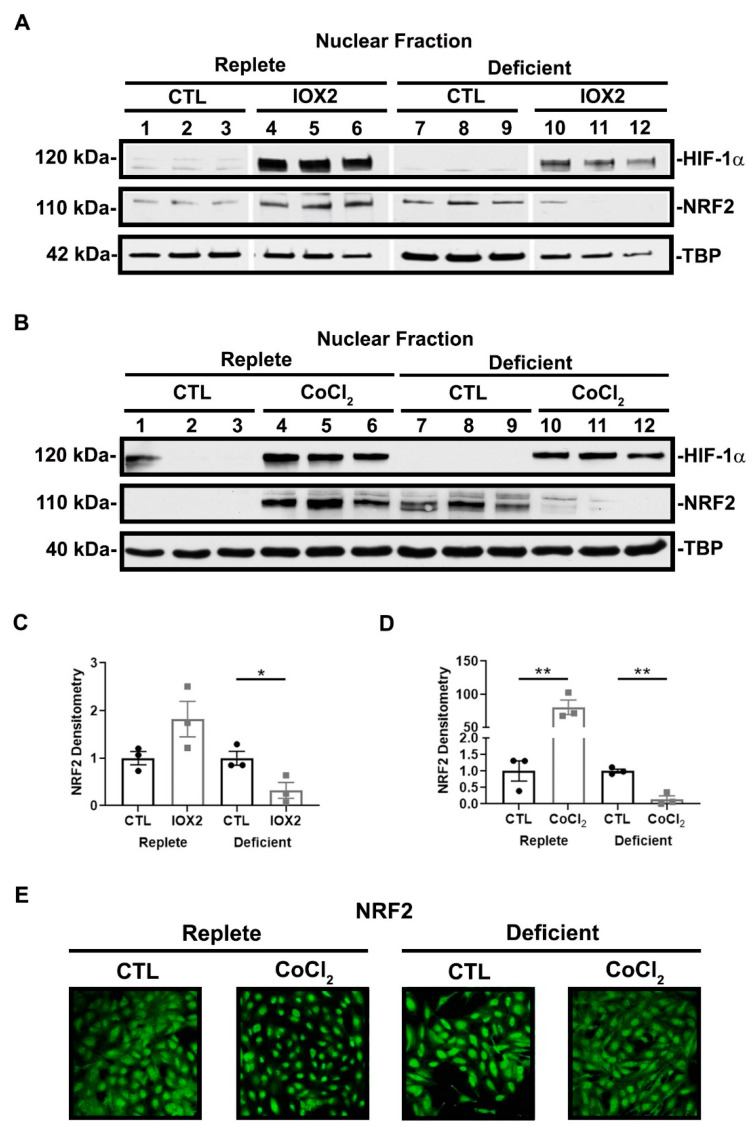
NRF2 nuclear localization is responsive to HIF-1α activation and nutrient availability. (**A**,**B**) Representative immunoblots of HIF-1α and NRF2 nuclear localization after 16 h exposure to nutrient-replete or nutrient-deficient conditions in the presence or absence of 50 μM IOX2 or 200 μM CoCl_2_. IOX2 and CoCl_2_ increase HIF-1α nuclear localization regardless of nutrient condition. In replete conditions, IOX2- and CoCl_2_-mediated HIF-1α activation increase NRF2 in the nuclear fraction of the cell lysates. Deficient conditions alone increase NRF2 nuclear localization but is suppressed in the presence of IOX2- and CoCl_2_-mediated HIF-1α activation. (**C**,**D**) Densitometry of NRF2 normalized to TBP. Bars are mean ± S.E.M. Unpaired Student’s *t*-test. * *p* < 0.05 and ** *p* < 0.01. (**E**) Representative immunofluorescent images of NRF2 protein expression after 16 h exposure to nutrient-replete or nutrient-deficient conditions in the presence or absence of 200 μM CoCl_2_. In replete conditions, NRF2 nuclear localization increases with CoCl_2_-mediated HIF-1α activation. However, in deficient conditions, CoCl_2_-mediated HIF-1α activation suppresses NRF2 nuclear localization.

To further explore the effect of HIF-1α activation and nutrient availability on NRF2 activity, we assessed NRF2 nuclear localization in the presence or absence of HIF-1α activators, IOX2 and CoCl_2_. As expected, both IOX2 and CoCl_2_ increased HIF-1α nuclear localization regardless of nutrient availability. In replete conditions, IOX2- and CoCl_2_-mediated HIF-1α activation increased NRF2 nuclear localization. However, although nutrient-deficient conditions alone increased NRF2 nuclear localization, this was suppressed in the presence of IOX2- and CoCl_2_-mediated HIF-1α activation (Figure 6A,B). Cytosolic levels of NRF2 remained unchanged in either nutrient condition in the presence or absence of HIF-1α activation (data not shown). To support our immunoblot findings, immunofluorescence staining for NRF2 revealed its nuclear localization occurring in replete conditions with CoCl_2_-mediated HIF-1α activation. Conversely, in deficient conditions, CoCl_2_-mediated HIF-1α activation suppressed NRF2 nuclear localization (Figure 6C). Collectively, these results are consistent with the likelihood that HIF-1α-mediated suppression of NRF2 activity in deficient or severe conditions is due to the lack of NRF2 nuclear localization.

**Figure 7:**
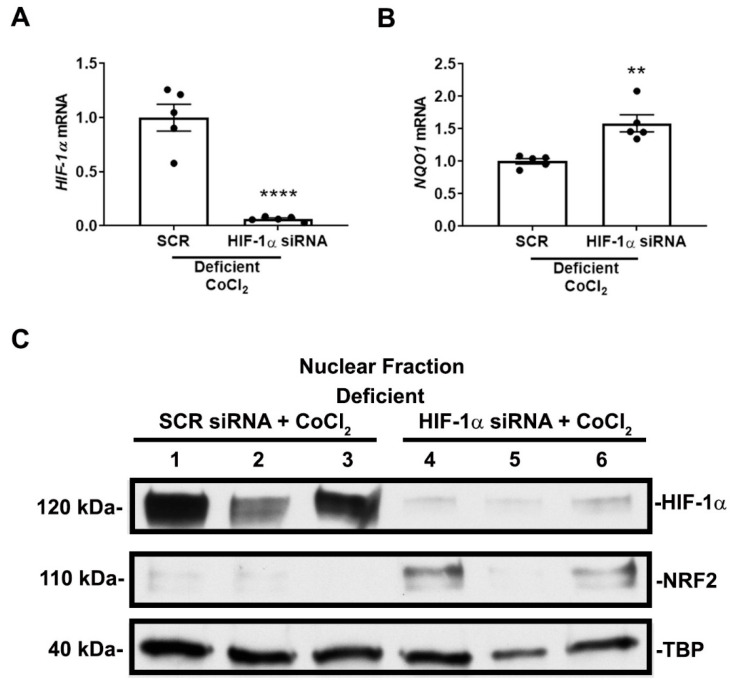
HIF-1α mediates NRF2 nuclear localization and activity in nutrient-deficient conditions. (**A**,**B**) *HIF-1α* and *NQO1* mRNA expression after siRNA transfection and exposure to 16 h of deficient conditions in the presence of 200 μM CoCl_2_. Transfection results in greater that 90% knockdown efficiency in *HIF-1α* mRNA expression. siRNA-mediated HIF-1α knockdown increases *NQO1* mRNA expression. (**A**,**B**) *n* = 5. Bars are mean ± S.E.M. Unpaired Student’s *t*-test. ** *p* < 0.01 and **** *p* < 0.0001. (**C**) Representative immunoblots of nuclear HIF-1α and NRF2 protein expression after siRNA transfection and exposure to 16 h of deficient conditions in the presence of 200 μM CoCl_2_. siRNA-mediated HIF-1α knockdown restores NRF2 nuclear localization. A second independent experiment was performed showing similar results.

To substantiate that HIF-1α mediates the suppression of NRF2 nuclear localization and activity in nutrient-deficient conditions, we performed siRNA-mediated HIF-1α knockdown prior to exposure to deficient conditions and CoCl_2_. Our method achieved greater than 90% knockdown efficiency (Figure 7A). Knocking down HIF-1α prior to exposure to deficient conditions and CoCl_2_ increased *NQO1* mRNA expression (Figure 7B) and restored NRF2 nuclear localization (Figure 7C, Supplemental Figure S2) suggesting HIF-1α mediates the suppression of NRF2 nuclear localization and activity in nutrient-deficient or severe conditions.

**Figure 8:**
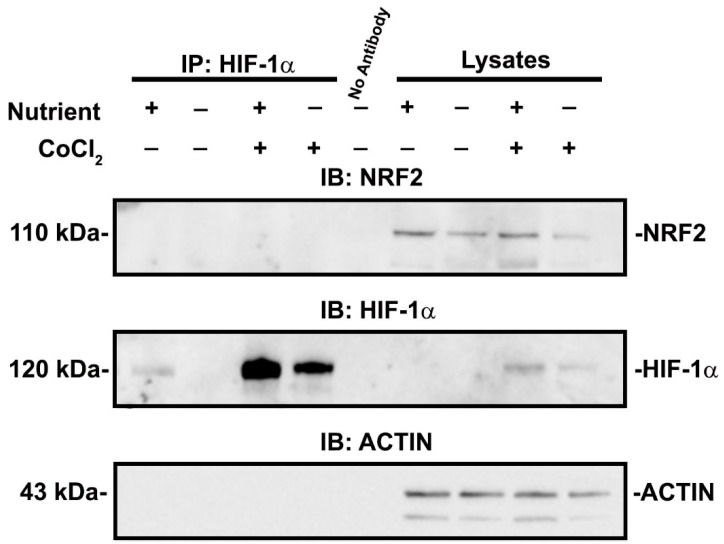
HIF-1α and NRF2 do not directly interact in cells exposed to experimental conditions. HIF-1α is immunoprecipitated and immunoblots for NRF2 and HIF-1α were performed after exposure to replete or deficient conditions in the presence or absence of 200 μM CoCl_2_. In all cell culture conditions, there was no indication of co-immunoprecipitation of HIF-1α and NRF2. Cell lysates without immunoprecipitation are shown as a control.

The effect of HIF-1α was not due to direct interaction because we could not co-immunoprecipitate HIF-1α and NRF2 in cells exposed to either nutrient condition in the presence or absence of CoCl_2_ (Figure 8A). Overall, our results demonstrate that HIF-1α influences NRF2 nuclear localization and target gene transcription in a manner that is dependent on nutrient availability.

## 4. Discussion

AKI remains a significant problem affecting hospitalized patients and contributes to the long-term risks of developing CKD and ESRD [6,49,50]. The development of CKD is often associated with the severity of AKI [51]. Prior investigations have identified tubular epithelial cell cycle arrest, microvascular rarefaction, and the development of interstitial fibrosis as contributors, but they are unlikely to be the only causes [52,53,54,55,56]. In this regard, we chose to study the relationship between NRF2 and HIF-1α, two well-known pathways involved in AKI-to-CKD pathogenesis.

HIF-1α is upregulated during IRI-AKI and activates pathways involved in vasculogenesis and glucose uptake and metabolism. HIF-1α expression is induced throughout the nephron including [57] in proximal tubular epithelial cells during the acute reperfusion phase of ischemic AKI [26,27,58]. As NRF2 has also been shown to be expressed in proximal tubules and other cortical nephron segments [59], it is possible that the HIF-1α and NRF2 pathways interact. Although it has been shown that short-term preischemic activation of HIF-1α reduces IRI severity, chronic activation may promote chronic kidney injury through inflammation, cell proliferation, and the development of fibrosis [60,61,62,63]. The downstream mediators of these opposing acute and chronic effects were not fully understood.

NRF2 is a cytoprotective pathway known to be protective in IRI-AKI. Genetic and pharmacologic NRF2 activation reduce AKI severity and prevent AKI-to-CKD progression [16,17,18]. Previously, we reported a maladaptive reduction in NRF2 activity in wild-type mice with severe IRI leading to progression to CKD [17]. Based on this result, we hypothesized that severity of IRI-AKI affects the expression of protective NRF2, which in turn affects long-term kidney outcomes.

To test this, we determined in our murine model system a threshold of IRI severity that leads to progressive kidney disease. While moderate and severe IRI had similar increases in serum creatinine and BUN at day 1, only the severe IRI mice exhibited persistent elevation in these functional markers (Figure 1A,B). As expected, the severe IRI mice also exhibited increased tubular injury markers *Kim1* and *Ngal* (Figure 1C,D) and renal fibrosis (Figure 2A,B).

We then examined how injury severity affected NRF2 activity. When assessing NQO1 protein, we found that all levels of injury acutely suppressed NQO1 at day 1. NQO1 eventually returned to baseline levels in mild IRI but continued to decline in severe IRI (Figure 3A,C). When we measured NQO1 expression, we found that mild IRI led to an early and sustained increase of NRF2 activity. Conversely, moderate and severe IRI led to delayed and transient increases of NRF2 activity (Figure 3D). Importantly, at day 1, severe IRI was associated with increased HIF-1α activity and decreased NRF2 activity (Figure 3D,E). This finding suggests that in severe IRI HIF-1α activation has a suppressive effect on NRF2 activity. Our results also indicate that mild IRI is associated with a more rapid recovery of NRF2 activity which may protect against AKI-to-CKD progression [17,18].

Our findings help to clarify existing studies that examine NRF2 in AKI. In Swiss mice, bilateral IRI increases *Nqo1* gene expression, but in CD-1 mice there was no change [15,64]. Whereas the latter study identified other increased NRF2 targets, *Nqo1* is more specific for NRF2 [13]. In C57BL/6 mice given unilateral ischemia for 23 min, NRF2 mRNA and protein expression increased at 4 and 8 h but not at 24 h after ischemia [65]. Decreased NRF2 activity was found during AKI-to-CKD progression in folic acid nephropathy in C57BL/6 mice and after kidney injury in humans [19]. Similarly, aristolochic acid-induced AKI in C57BL/6 mice showed impaired NRF2 activation and expression of target genes [66]. Considering these different responses, it is possible that the type of AKI and severity of injury affects overall NRF2 responses.

It is known that the IRI model is affected by ischemia time, clamp type, surgical technique, mouse strain, surgeon/laboratory, and whether the injury is unilateral or bilateral [48]. In our unilateral IRI model, we varied only ischemia time to titrate the injury while keeping other variables constant. It is possible that prior studies showing NRF2 activation in AKI utilized mild injury, whereas studies showing decreases in NRF2 activity utilized more severe injury. Our results would suggest this is the case.

To examine the relationship between NRF2 and HIF-1α, we induced HIF-1α activation in mice with IOX2. IOX2-mediated HIF-1α activation decreased NRF2 target genes suggesting that HIF-1α mediates NRF2 activity (Figure 4A,B). In our IRI model, we showed an inverse relationship between NRF2 and HIF-1α activities during recovery from different IRI severities. To further investigate this, we mimicked mild and severe ischemia conditions in vitro in HK-2 cells by using nutrient-replete or nutrient-deficient conditions, respectively. We then activated HIF-1α with CoCl_2_ or IOX2. In nutrient-replete conditions, HIF-1α activation enhanced NRF2 nuclear localization and target gene transcription, but in nutrient-deficient conditions, NRF2 nuclear localization and activity was suppressed (Figure 5A–E and Figure 6A–C). This latter effect was directly dependent on HIF-1α because HIF-1α siRNA knockdown restored NRF2 nuclear localization and target gene transcription (Figure 7B,C). A limitation of our study is that we did not perform our experiments in primary tubular cell lines. Nonetheless, our current results in HK-2 cells help to explain the results of our animal studies.

We also did not assess the von Hippel–Lindau (VHL) protein, which participates in the degradation of HIF-1α. It has been found that VHL deletion (and increased HIF1-α activity) in tubular cells can provide protection against AKI due to IRI and rhabdomyolysis [57,67]. Similar to the results we report above, we would predict that nutrient environment, or severity of kidney injury, would alter the effect of VHL deletion on HIF-1α-mediated regulation of NRF2 in our model. Notably, the prior studies in animals do not specifically assess injury severity.

It is presently unclear exactly how HIF-1α affects NRF2, although we ruled out a direct interaction (Figure 8). NRF2 undergoes KEAP1-independent regulation via protein kinase C (PKC), c-Jun N-terminal kinase (JNK), phosphatidylinositol 3-kinase/protein kinase B (PI3K/AKT), casein kinase 2 (CK2), AMP activated protein kinase (AMPK), extracellular signal regulated kinase (ERK), and glycogen synthase kinase 3β (GSK3β), among other pathways [19,64,68,69,70,71,72,73]. NRF2 may also be affected by activity of importins, which provide access to the nucleus [74]. Further studies are needed to determine the mechanism of effect.

The timing of NRF2 and HIF-1α activation are critical to the overall renoprotective effect. Prior studies show that preconditioning or early activation of the NRF2 or HIF-1α response ameliorates injury, whereas activation at later timepoints does not afford protection [16,18,62]. We demonstrate that more severe IRI in mice leads to higher HIF-1α and lower NRF2 activity at day 1. Early stages of AKI injury are critical time points at which NRF2 is required to protect against further injury [16]. The suppression of NRF2 could be a contributor to the AKI-to-CKD progression observed in severe injuries.

## 5. Conclusions

We demonstrate that severe AKI leading to CKD and fibrosis is associated with a maladaptive suppression of the protective NRF2 response. In vivo, this was associated with stronger HIF-1α activation. We show that in kidney cells in vitro, HIF-1α suppresses NRF2 activity under stringent nutrient-deficient conditions that would mimic severe AKI. We propose that the higher HIF-1α activity induced by severe injury leads to the suppression of NRF2, which predisposes the kidney to chronic fibrosis.

## Data Availability

All of the data is contained within the article and the supplementary materials.

## Supplementary Materials

The following supporting information can be downloaded at: https://www.mdpi.com/article/10.3390/antiox11091810/s1, Figure S1: Longer ischemia times lead to more fibronectin accumulation. Mice were exposed to the indicated ischemia times and euthanized at 10 days. Fibronectin was assessed by western blot in Figure 2, with densitometry performed and presented.; Figure S2: HIF-1alpha knockdown increases NRF2 nuclear localization. Shown is densitometry from western blots assessing nuclear localization of NRF2 in HK-2 cells exposed to nutrient-deficient conditions and CoCl_2_. Two independent experiments were combined to generate this figure.
