# Supplementary material for: Suppression of NRF2 Activity by HIF-1α Promotes Fibrosis after Ischemic Acute Kidney Injury
Source: Antioxidants (Basel). 2022 Sep 14;11(9):1810. doi: 10.3390/antiox11091810 (PMC9495756; doi:10.3390/antiox11091810)
Supplement: Supplementary file 1 [file antioxidants-11-01810-s001.zip › antioxidants-1892747-supplementary.pdf]

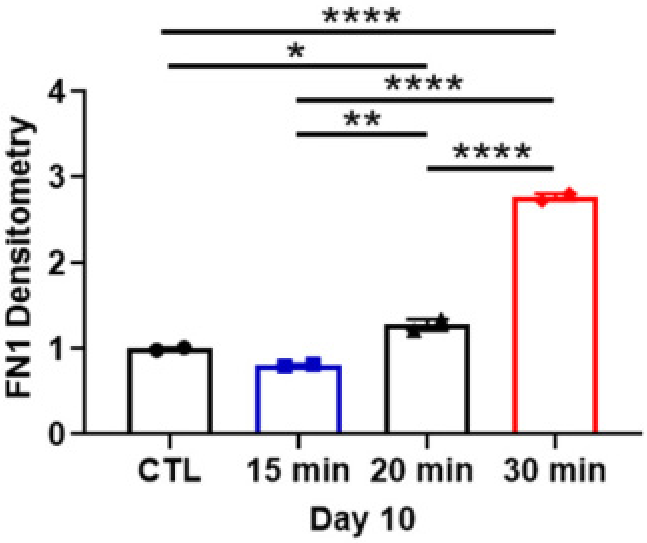

Figure S1. Longer ischemia times lead to more fibronectin accumulation. Mice were exposed to the indicated ischemia times and euthanized at 10 days. Fibronectin was assessed by western blot in Figure 2, with densitometry performed and presented. * $p < 0.05$, ** $p < 0.01$, **** $p < 0.0001$.

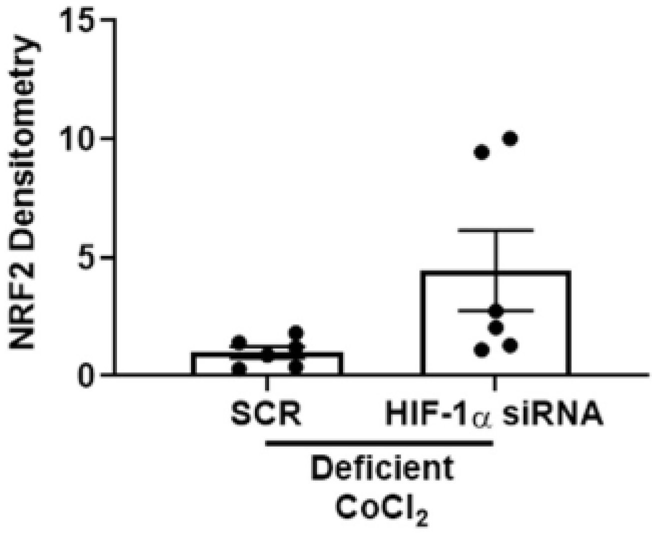

Figure S2. HIF-1alpha knockdown increases NRF2 nuclear localization. Shown is densitometry from western blots assessing nuclear localization of NRF2 in HK-2 cells exposed to nutrient-deficient conditions and $CoCl_2$. Two independent experiments were combined to generate this figure.
